# Acute Alithiasic Cholecystitis and Human Herpes Virus Type-6 Infection: First Case

**DOI:** 10.1155/2016/9130673

**Published:** 2016-04-20

**Authors:** Maria Miguel Gomes, Henedina Antunes, Ana Luísa Lobo, Fernando Branca, Jorge Correia-Pinto, João Moreira-Pinto

**Affiliations:** ^1^Department of Pediatrics, Hospital de Braga, Sete Fontes, São Victor, 4710-243 Braga, Portugal; ^2^Gastroenterology, Hepatology and Nutrition Unit, Department of Pediatrics, Hospital de Braga, Sete Fontes, São Victor, 4710-243 Braga, Portugal; ^3^Life and Health Sciences Research Institute (ICVS), School of Health Sciences, University of Minho, 4710-057 Braga, Portugal; ^4^ICVS/3B's-PT Government Associate Laboratory, Braga/Guimarães, Portugal; ^5^Department of Pediatrics, Hospital de Alto Ave, 4835-044 Guimarães, Portugal; ^6^Department of Clinical Pathology, Hospital de Braga, Sete Fontes, São Victor, 4710-243 Braga, Portugal; ^7^Department of Pediatric Surgery, Hospital de Braga, Sete Fontes, São Victor, 4710-243 Braga, Portugal

## Abstract

A three-year-old male child presented with erythematous maculopapular nonpruritic generalized rash, poor feeding, vomiting, and cramping generalized abdominal pain. He was previously healthy and there was no family history of immunologic or other diseases. On examination he was afebrile, hemodynamically stable, with painful palpation of the right upper quadrant and positive Murphy's sign. Laboratory tests revealed elevated inflammatory markers, elevated aminotransferase activity, and features of cholestasis. Abdominal ultrasound showed gallbladder wall thickening of 8 mm with a positive sonographic Murphy's sign, without gallstones or pericholecystic fluid. Acute Alithiasic Cholecystitis (AAC) was diagnosed. Tests for underlying infectious causes were negative except positive blood specimen for Human Herpes Virus Type-6 (HHV-6) by polymerase chain reaction. With supportive therapy the child became progressively less symptomatic with gradual improvement. The child was discharged on the sixth day, asymptomatic and with improved analytic values. Two months later he had IgM negative and IgG positive antibodies (1/160) for HHV-6, which confirmed the diagnosis of previous infection. In a six-month follow-up period he remains asymptomatic. To the best of our knowledge, this represents the first case of AAC associated with HHV-6 infection.

## 1. Introduction

Acute Alithiasic Cholecystitis (AAC) is defined as an acute necroinflammatory disease of the gallbladder in the absence of cholelithiasis [1, 2]. It accounts for approximately 2–15% of all cases of acute cholecystitis [3].

The pathogenesis of AAC is multifactorial and likely results from bile stasis, ischemia, or both [1–3]. Bile stasis can be caused by fasting, obstruction, postsurgical or procedural irritation, or ileus, which can lead to bile inspissation that is directly toxic to the gallbladder epithelium [3]. Ischemia may occur from systemic inflammation and could have deleterious effects directly to all layers of the gallbladder wall [3]. Various conditions predispose to its occurrence: trauma, recent surgery, shock, burns, total parenteral nutrition, chronic diseases, and viral (hepatotropic virus) or bacterial (mostly gram-negative or anaerobic) infections [1–3].

The diagnosis of AAC is difficult and relies on combination of clinical history, physical examination, laboratory tests, and radiologic image [1–6].

Treatment depends on the time of diagnosis. In early stages, conservative treatment is sufficient [2–7]. Surgical procedures like cholecystectomy should be reserved for patients with complications [2–7].

Complications of AAC may occur in about 40% of the cases (gangrene, perforation, and empyema) [1–3]. Mortality depends on the underlying medical condition, ranging from 90% in critically ill patients up to 10% in the outpatient [1–3].

## 2. Case Description

A three-year-old male child presented with a two-day history of erythematous maculopapular nonpruritic generalized rash and a one-day history of malar erythema, poor feeding, vomiting, and abdominal pain. He was previously healthy and there was no family history of immunologic or other diseases. The child did not have a previous or recent history of exanthem subitum. There was not a similar exposure in his family members. On examination he was afebrile, hemodynamically stable, with painful palpation of the right upper quadrant and positive Murphy's sign. He had no lymphadenopathy or hepatosplenomegaly. The rest of the examination was unremarkable. Laboratory tests revealed elevated inflammatory markers (C-reactive protein (CRP) 55.6 mg/L, reference range (RR) < 3.0), elevated aminotransferase activity (alanine aminotransferase (ALT) 727 U/L, RR 12–78, aspartate aminotransferase (AST) 717 U/L, RR < 50), and features of cholestasis (g-glutamyl transferase (GGTP) 364 U/L, RR 4–22, alkaline phosphatase (ALP) 536 U/L, RR < 362, and direct bilirubin 0.34 mg/dL,RR 0.0–0.2). Blood coagulation tests and total bilirubin were normal (Table 1). Abdominal ultrasound showed gallbladder wall thickening of 8 mm with a positive sonographic Murphy's sign, without gallstones or pericholecystic fluid. An AAC was diagnosed. Hospitalization was decided for intravenous fluids and clinical and analytic monitorization. Tests for underlying causes of AAC, including Human Immunodeficiency Virus (HIV), Hepatitis A Virus (HAV), toxoplasmosis, Epstein-Barr Virus (EBV), Cytomegalovirus (CMV), Parvovirus B19, and* Mycoplasma pneumonia,* were negative. Blood specimen deoxyribonucleic acid (DNA) for Human Herpes Virus Type-6 (HHV-6) by polymerase chain reaction was positive. Serologic tests for hepatitis B and hepatitis C were not done because maternal third-trimester serology was negative.

The child became progressively less symptomatic with gradual improvement. Forty-eight hours later ultrasonographic examination showed decreased wall thickening. The child was discharged on the sixth day, asymptomatic and with improved analytic values: CRP 5.31 mg/L, ALT 202 U/L, AST 51 U/L, GGTP 389 U/L, ALP 484 U/L, and direct bilirubin < 0.10 mg/dL. There was no need for treatment with antibiotics, antiviral, or parenteral nutrition. One month later he had normal values for inflammatory markers and normal aminotransferase activity and no cholestasis. Two months later he had IgM negative and IgG positive antibodies (1/160) for HHV-6, which confirmed the diagnosis of previous infection. In a six-month follow-up period he remains asymptomatic.

## 3. Discussion

To the best of our knowledge, this represents the first case of AAC associated with HHV-6 infection.

HHV-6 is ubiquitous beta herpes virus [8]. It exhibits a wide cell tropism in vivo and induces a lifelong latent infection in humans. The diagnosis of HHV-6 infection is performed by both serologic and direct methods [8, 9]. The most prominent technique is the quantification of viral DNA in blood, other body fluids, and tissues by means of real-time polymerase chain reaction (PCR) [8, 9]. Many active HHV-6 infections are asymptomatic and correspond to primary infections, reactivations, or exogenous reinfections. However, HHV-6 may be the cause of serious diseases, particularly in immunocompromised. An emblematic example of HHV-6 pathogenicity is exanthema subitum (also known as roseola infantum or sixth disease), a generally benign febrile exanthem of infancy, associated with primary infection. Treatment is supportive and recovery is usually complete with no significant sequelae [9]. The antiviral compounds ganciclovir, foscarnet, and cidofovir are effective against active HHV-6 infections, but the indications for treatment, as well as the conditions of drug administration, are not formally approved to date [8].

Gallbladder disease is rare in the pediatric population [1–3, 10]. AAC accounts for 30–50% of pediatric cholecystitis cases [10]. In most cases, AAC in children occurs during the course of infectious diseases. AAC caused by viral infection is extremely rare in the pediatric population. Ultrasonography is the main diagnostic radiologic exam of acute cholecystitis (efficiency is approximately 90%) [11, 12]. Criteria such as gallbladder wall thickening over 3 mm, gallbladder distention, localized tenderness, pericholecystic fluid, and sludge have been used for the diagnosis. A combination of at least two of the above-mentioned findings is considered to indicate AAC. Generally patients present with similar clinical manifestations: fever, nausea, vomiting, and pain in the right upper quadrant. There are several reported cases of acute hepatotropic virus infection (Epstein-Barr Virus, hepatitis A, B, and C virus) and cholestatic hepatitis [13–17]. In our patient the cholestatic hepatitis was probably due to HHV-6 infection. Quantification of HHV-6 DNA in blood was positive and confirmed the acute infection. Also two months later serologic test confirmed the HHV-6 infection. The treatment of uncomplicated ACC is conservative and symptomatic, which was done in our patient. It consists of the application, according to needs, of analgesics, antiemetics, antibiotics, fluid therapy, and parental nutrition. Abdominal Computerized Tomography Scan (CT Scan) is reserved for cases of suspected complications.

AAC is difficult to diagnose, but an early correct assessment is essential to successful treatment. Clinicians should be aware of the possible involvement of the gallbladder during HHV-6 infection to avoid unnecessary invasive procedures or overuse of antibiotics.

## Figures and Tables

**Table 1:** Laboratory tests at admission.

Parameters at admission	Values	Reference range
*Hematologic values*		
Hemoglobin	12.1 g/dL	11.5–13.5
Hematocrit	34.3%	34.0–40.0
Mean corpuscular volume	76.2 fL	75.0–87.0
Mean corpuscular hemoglobin	35.3 g/dL	31.0–37.0
Leukocytes	10.3 × 10^3^/*μ*L	5.0–14.5
Neutrophils	6.9 × 10^3^/*μ*L	1.0–8.5
Lymphocytes	1.8 × 10^3^/*μ*L	1.5–8.0
Platelets	341 × 10^3^/*μ*L	200–450
*Blood coagulation tests*		
Activated partial thromboplastin Time	34.4 seconds	25.0–35.0
Prothrombin time	14.0 seconds	8.0–14.0
International normalized ratio	1.0	0.1–1.2
*Biochemistry*		
Urea	24 mg/dL	15–39
Creatinine	0.3 mg/dL	0.31–0.42
Sodium	137 mmol/L	136–145
Potassium	4.0 mmol/L	3.5–5.1
Chlorides	103 mmol/L	98–107
Amylase	26 U/L	5–65
Lipase	64 U/L	73–393
Total bilirubin	0.62 mg/dL	0.1–1.0
Direct bilirubin ↑	0.34 mg/dL	0.0–0.2
ALT ↑	717 U/L	12–78
AST ↑	727 U/L	<50
GGTP ↑	364 U/L	4–22
ALP ↑	536 U/L	<362
CRP ↑	55.6 mg/L	<3.0
*Polymerase chain reaction*		
HHV-6	*Positive*	

2 months later	Values	Reference range

HHV-6 Atc IgM	Negative	<1/20
HHV-6 Atc IgG	*Positive 1/160*	*<1/80*

ALP: alkaline phosphatase; ALT: alanine aminotransferase; AST: aspartate aminotransferase; CRP: C-reactive protein; GGTP: g-glutamyl transferase.

## Abbreviations

AAC: Acute Alithiasic Cholecystitis
DNA: Deoxyribonucleic acid
HHV-6: Human Herpes Virus Type-6
RR: Reference range.

## References

[1] Barie P. S., Eachempati S. R. (2003). Acute acalculous cholecystitis. *Current Gastroenterology Reports*.

[2] Owen C. C., Jain R. (2005). Acute acalculous cholecystitis. *Current Treatment Options in Gastroenterology*.

[3] Huffman J. L., Schenker S. (2010). Acute acalculous cholecystitis: a review. *Clinical Gastroenterology and Hepatology*.

[4] Mariat G., Mahul P., Prévôt N. (2000). Contribution of ultrasonography and cholescintigraphy to the diagnosis of acute acalculous cholecystitis in intensive care unit patients. *Intensive Care Medicine*.

[5] Babb R. R. (1992). Acute acalculous cholecystitis: a review. *Journal of Clinical Gastroenterology*.

[6] Glenn F. (1979). Acute acalculous cholecystitis. *Annals of Surgery*.

[7] Imamoglu M., Sarihan H., Sari A., Black C. T., Lally K. P., Andrassy R. (2002). Acute acalculous cholecystitis in children: diagnosis and treatment. *Journal of Pediatric Surgery*.

[8] Agut H., Bonnafous P., Gautheret-Dejean A. (2015). Laboratory and clinical aspects of human herpesvirus 6 infections. *Clinical Microbiology Reviews*.

[9] Stone R. C., Micali G. A., Schwartz R. A. (2014). Roseola infantum and its causal human herpesviruses. *International Journal of Dermatology*.

[10] Tsakayannis D. E., Kozakewich H. P. W., Lillehei C. W. (1996). Acalculous cholecystitis in children. *Journal of Pediatric Surgery*.

[11] Deitch E. A., Engel J. M. (1981). Acute acalculous cholecystitis: ultrasonic diagnosis. *The American Journal of Surgery*.

[12] Becker C. D., Burckhardt B., Terrier F. (1986). Ultrasound in postoperative acalculous cholecystitis. *Gastrointestinal Radiology*.

[13] Alkhoury F., Diaz D., Hidalgo J. (2015). Acute acalculous cholecystitis (AAC) in the pediatric population associated with Epstein–Barr Virus (EBV) infection. Case report and review of the literature. *International Journal of Surgery Case Reports*.

[14] Agergaard J., Larsen C. S. (2015). Acute acalculous cholecystitis in a patient with primary Epstein-Barr virus infection: a case report and literature review. *International Journal of Infectious Diseases*.

[15] Kim A., Yang H. R., Moon J. S., Chang J. Y., Ko J. S. (2014). Epstein-barr virus infection with acute acalculous cholecystitis. *Pediatric Gastroenterology, Hepatology & Nutrition*.

[16] Kaya S., Eskazan A. E., Ay N. (2013). Acute acalculous cholecystitis due to viral hepatitis A. *Case Reports in Infectious Diseases*.

[17] Trésallet C., Bastien L., Rabahi Y., Cadi M., Leroux G., Menegaux F. (2010). Hepatitis C virus infection revealed by an acute acalculous cholecystitis. *Journal de Radiologie*.

